# Bcl-6-dependent risk stratification by nuclear expression of Peli1 in diffuse large B-cell lymphoma

**DOI:** 10.7150/jca.67569

**Published:** 2022-11-14

**Authors:** Ki Rim Lee, Jeong-Ok Lee, Jong Seok Lee, Jin Ho Paik

**Affiliations:** 1 Department of Pathology, Seoul National University Bundang Hospital, Seongnam, South Korea; 2 Department of Pathology, Seoul National University College of Medicine, Seoul, South Korea; 3 Department of Internal Medicine, Seoul National University Bundang Hospital, Seongnam, South Korea; 4 Department of Internal Medicine, Seoul National University College of Medicine, Seoul, South Korea

**Keywords:** malignant lymphoma, diffuse large B-cell lymphoma, Bcl-6, Peli1

## Abstract

**Background/Aim:** Peli1 is an E3 ubiquitin ligase involving lymphomagenesis by lysine 63 ubiquitination-mediated stabilization of Bcl-6 with in diffuse large B-cell lymphoma (DLBCL).

**Materials and Methods:** We categorized nuclear expression of Peli1 according to Bcl-6 status by immunohistochemistry in DLBCL (n=100), and analyzed clinicopathologic association with prognosis.

**Results:** We established Bcl-6/Peli1 risk model composed of high risk (Bcl-6+/Peli1+ or Bcl-6-/Peli1-; n=64) and low risk (Bcl-6+/Peli1- or Bcl-6-/Peli1+; n=36). High risk group had more frequent non-GCB subtype (83% vs 64%; p=0.033) and Bcl-6-negativity (69% vs 28%; p<0.001) than low risk group. Univariate survival analysis for progression-free survival (PFS) and overall survival (OS) revealed Bcl-6/Peli1 risk group (p=0.026 and p=0.021) and other conventional variables including international prognostic index (IPI), stage, ECOG performance status, number of extranodal sites were significant prognostic factors, along with B symptoms for OS. In multivariate analysis for PFS, Bcl-6/Peli1 risk group (p=0.032; HR=3.29), IPI (p=0.013; HR=3.39) and ECOG PS (p=0.035; HR=3.08) were independent prognostic factors. In multivariate analysis for OS, Bcl-6/Peli1 risk group (p=0.048; HR=7.87) and IPI (p=0.001; HR=12.15) were associated with prognosis.

**Conclusions:** DLBCL had distinctive risk groups according to pairs of nuclear Peli1 and Bcl-6 expression. These results suggest the potential role of Peli1 and Bcl-6 in risk assessment in DLBCL.

## Introduction

Diffuse large B cell lymphoma (DLBCL) is a heterogeneous clinicopathologic entity composed of neoplastic large B cells with germinal center B cell-like (GCB) or non-GCB/activated B cell-like (ABC) molecular features 1. While standard first-line immunochemotherapy with rituximab, cyclophosphamide, doxorubicin, vincristine and prednisone (R-CHOP) have good response in patients with DLBCL, about 1/3 or more patients remain refractory or experience relapse 2. Therefore, it is necessary to develop risk assessment model to predict outcome of DLBCL patients. Notably, many molecular markers have been developed for this purpose on the basis of oncogenic mechanism and 'cell of origin' with various feasibilities 3.

Peli1 is a type of E3 ubiquitin ligase with RING (really interesting new gene) domain 4. It has been known to play dual roles in biologic processes. It facilitates degradation of target protein via lysine 48 (K48) ubiquitination or stabilizes target protein via lysine 63 (K63) ubiquitination in a context-dependent manner 5. In experimental models, transgenic mice overexpressing Peli1 developed B cell lymphoma and/or lupus-like autoimmune disease with high density of B cell aggregate 6, 7, suggesting its strong association in B cell lymphomagenesis and B cell-related autoimmunity. So far, clinical studies for use of Peli1 as a prognostic marker in various tumors have rarely been reported with various results according to target molecules 6, 8, 9. In a few studies with tumor tissue samples of DLBCL with survival analysis, high level expression or nuclear-weighted expression of Peli1 showed poor prognosis, while its prognostic effects were limited in multivariate analysis in R-CHOP-treated cohort 6, 8. Since Peli1 exerts its oncogenic role by K63 ubiquitination of Bcl-6, an important driver molecule of DLBCL 6, we hypothesized that Peli1 may have a distinct clinical significance according to Bcl-6 status on the basis of molecular oncogenic mechanism. We explored the clinicopathologic and prognostic implication of Peli1 expression by dissecting it with Bcl-6 status. In the present study, we constructed a new risk model with Peli1 and Bcl-6 for assessing DLBCL patients.

## Materials and Methods

### Patients and Samples

In this study, we retrospectively included the cases of diffuse large B cell lymphoma, not otherwise specified (DLBCL, NOS) by the criteria of the revised 4th edition of WHO classification of hematopoietic and lymphoid neoplasm (2016 WHO classification), as shown in supplementary figure 1 10.

Briefly, we searched the database of pathology reports for the cases diagnosed at Seoul National University Bundang Hospital (SNUBH) as malignant lymphoma between May 2003 and January 2013. The cases were reviewed with pathological reports, histologic slides and molecular tests to specify the entity according to 2016 WHO classification, and the following cases were included: 1) the cases fitting the criteria of *de novo* DLBCL according to 2016 WHO classification, and 2) the cases with available paraffin blocks with sufficient residual tumor tissues. In addition, the following cases were excluded: 1) the cases with poor fixation or necrosis, 2) specific entities of large B cell lymphomas including 'EBV-positive DLBCL', 'primary mediastinal large B cell lymphoma', 'primary DLBCL of the central nervous system', 3) so-called 'gray-zone lymphomas', and 4) relapsed and/or transformed DLBCLs. In this way, a total of 100 cases of DLBCL were enrolled in this study.

The pathologic review process was performed by two pathologists (KRL and JHP) with histologic slides as well as immunohistochemical staining and molecular tests including EBV *in situ* hybridization and B cell clonality by using IgH gene rearrangement. All cases were positive for CD20 and 'cell of origin' classification including germinal center B cell-like (GCB) and non-GCB subtypes was performed by Hans algorithm 10, 11. Medical records were reviewed and clinical variables including Ann Arbor stage, international prognostic index (IPI) were retrieved for clinicopathologic analysis. This study was approved by the Institutional Review Board of Seoul National University Bundang Hospital (B-1306-208-301) and informed consent was waived due to the retrospective design using archived material in this study.

### Manufacturing tissue microarray, immunohistochemical staining, EBV *in situ* hybridization

Tumor tissues of round core with a diameter of 2 mm were sampled at well-fixed viable areas, and reconstructed into tissue microarray (TMA) blocks at Superbiochip (Seoul, South Korea) as previously described 12. Peli1 antibody was purchased from Santa Cruz Biotechnology (F7; 1: 50; Santa Cruz Biotechnology, Dallas, TX, USA). Immunohistochemistry for Peli1 was performed by manual method (microwave 5 min x3). Benchmark XT and Benchmark ULTRA (Roche Diagnostics, Basel, Swiss) were used for immunohistochemical staining for other antibodies and EBV *in situ* hybridization (EBV-ISH). For immunophenotyping of DLBCL, immunostainings for Bcl-6 (std32, ready-to-use, Ventana Medical Systems, Oro Valley, AZ, USA), CD10 (std32, ready-to-use, Ventana), MUM1 (mild32, 1:150, Dako, Glostrup, Denmark), and Bcl-2 (std32, 1:50, Dako) were performed by using Ventana autostainers (Benchmark XT and Benchmark ULTRA). For interpretation of 'cell of origin' immunostaining markers including Bcl-6, CD10 and MUM1, cut-off criteria of 30% was used 11, while Bcl-2 was regarded as positive if 50% of tumor cells were stained. EBV-ISH was also performed by using Ventana autostainer, and EBV-positive cases were excluded from this study. Interpretation was performed by two pathologists (KRL and JHP). Considering the previous study of nuclear weighted interpretation method 6 and mechanistic assumption that Peli1 participating in Bcl-6 stabilization would be located in nucleus, we simplified criteria of Peli1 expression as nuclear expression of > 30% of tumor cells, regardless of cytoplasmic staining.

### Statistical analysis

All statistical analysis was performed by using SPSS Statistics ver.19 (IBM, Chicago, IL). For clinicopathologic comparison, Chi square and Fisher's exact test were used. Univariate survival analysis was performed by using Kaplan-Meier analysis with log-rank test for statistical significance. Cox proportional hazard model was used for multivariate survival analysis with forward stepwise (conditional likelihood ratio) method incorporating all significant variables in univariate analysis. Overall survival (OS) was defined as the time interval from the date of diagnosis to the date of last follow-up or death. Progression-free survival (PFS) was defined as the time interval from the date of treatment to the date of progression with radiologic confirmation using computed tomography (CT) and/or positron emission tomography (PET)-CT after treatment or the date of death. All p values reported were two-sided. P values of less than 0.05 were regarded as statistically significant.

## Results

### Clinicopathologic characteristics of DLBCL

Clinicopathologic characteristics were summarized in Table 1. Briefly, male patients were slightly common (57%) with roughly equal distribution of age (>60, 52%). With similar distribution of stage (III-IV, 48%) and lactate dehydrogenase (elevated, 49%), cases with high IPI and extranodal sites (≥2) accounted for only 35% and 27%. Non-GCB subtype was predominant (76%).

### Expression patterns of Peli1 according to Bcl-6 and building a risk model of Bcl-6/Peli1 based on clinicopathologic features

**Figure 1:**
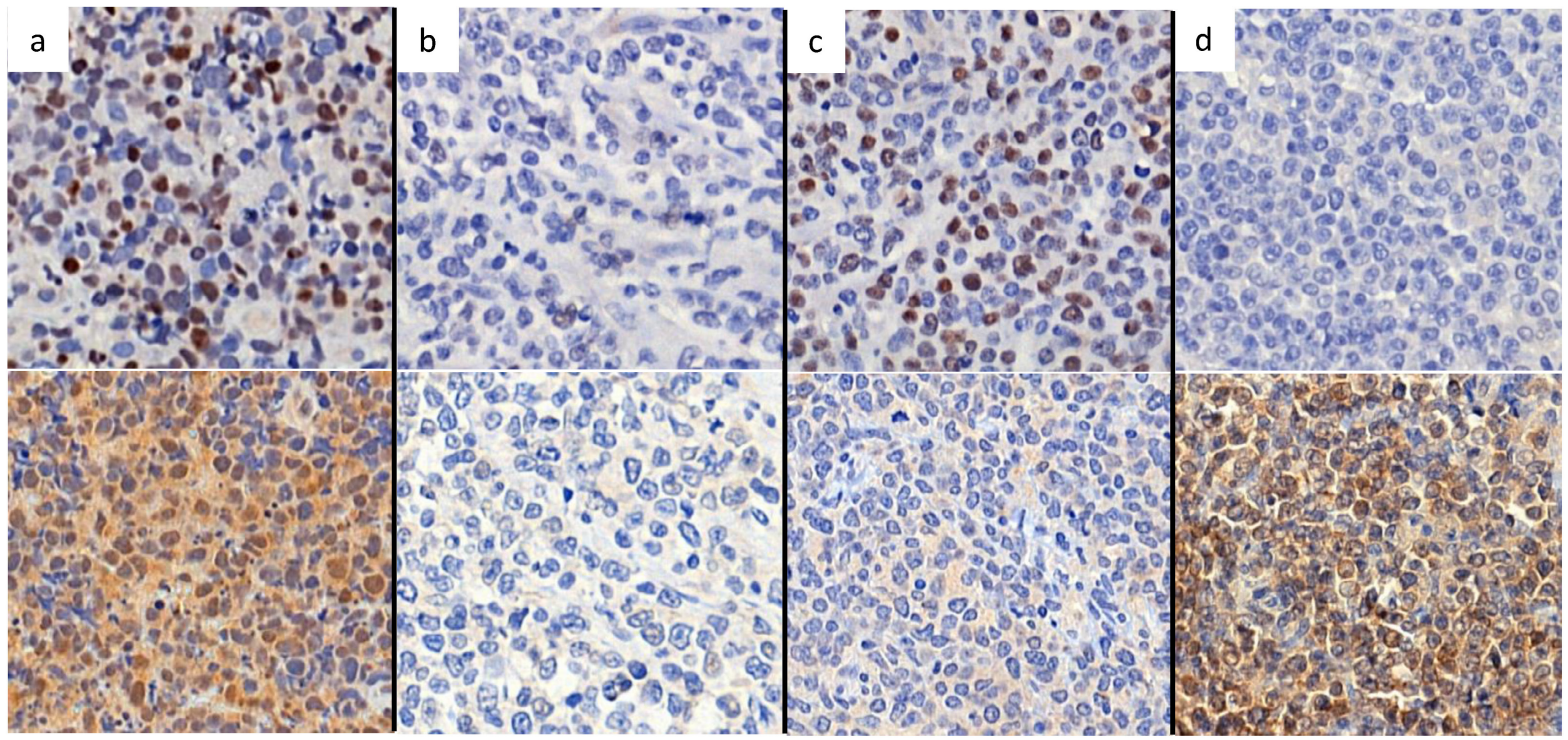
Representative figures of nuclear staining patterns of Bcl-6 and Peli1 by immunohistochemistry. When nuclei are stained as dark brown color contrasting with background, the case was interpreted as positive regardless of cytoplasmic stating. Figures on upper line show Bcl-6 status and figures on lower line show Peli1 status. (a) Bcl-6+, Peli1+, (b) Bcl-6-, Peli1- (c) Bcl-6+ Peli1- and (d) Bcl-6- Peli1+.

**Figure 2:**
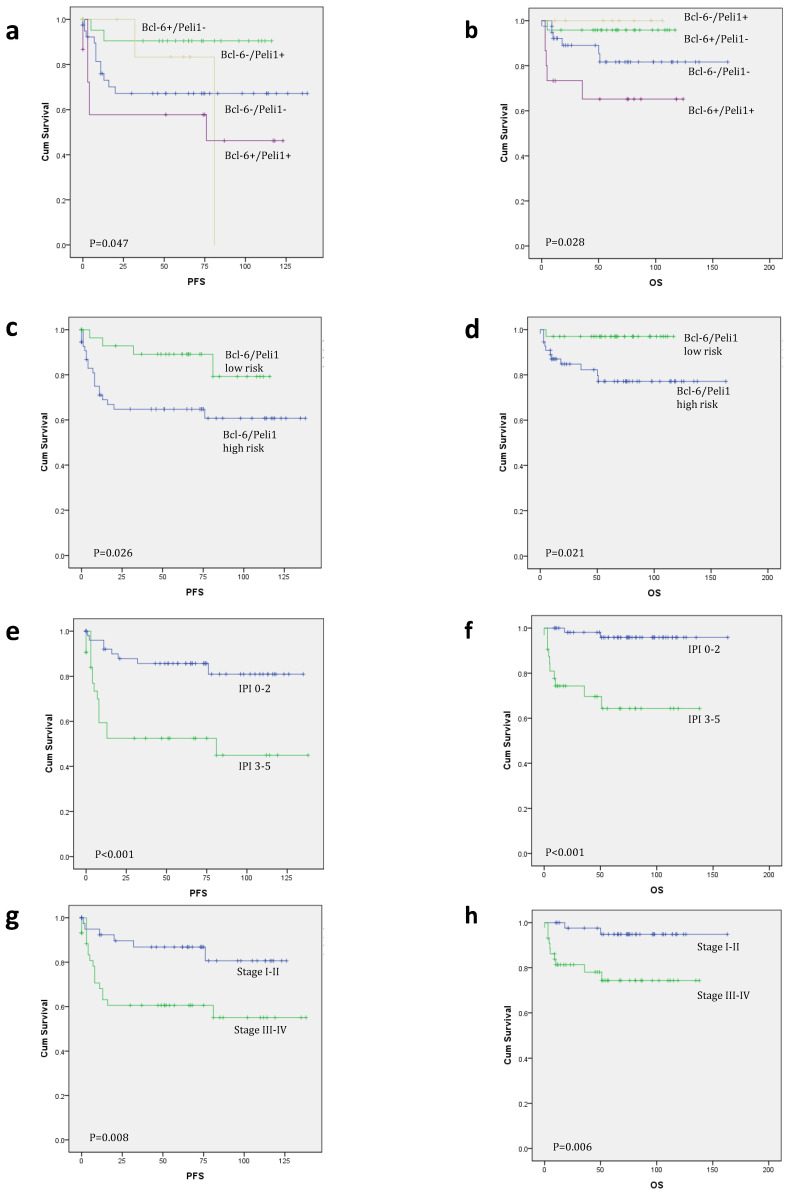

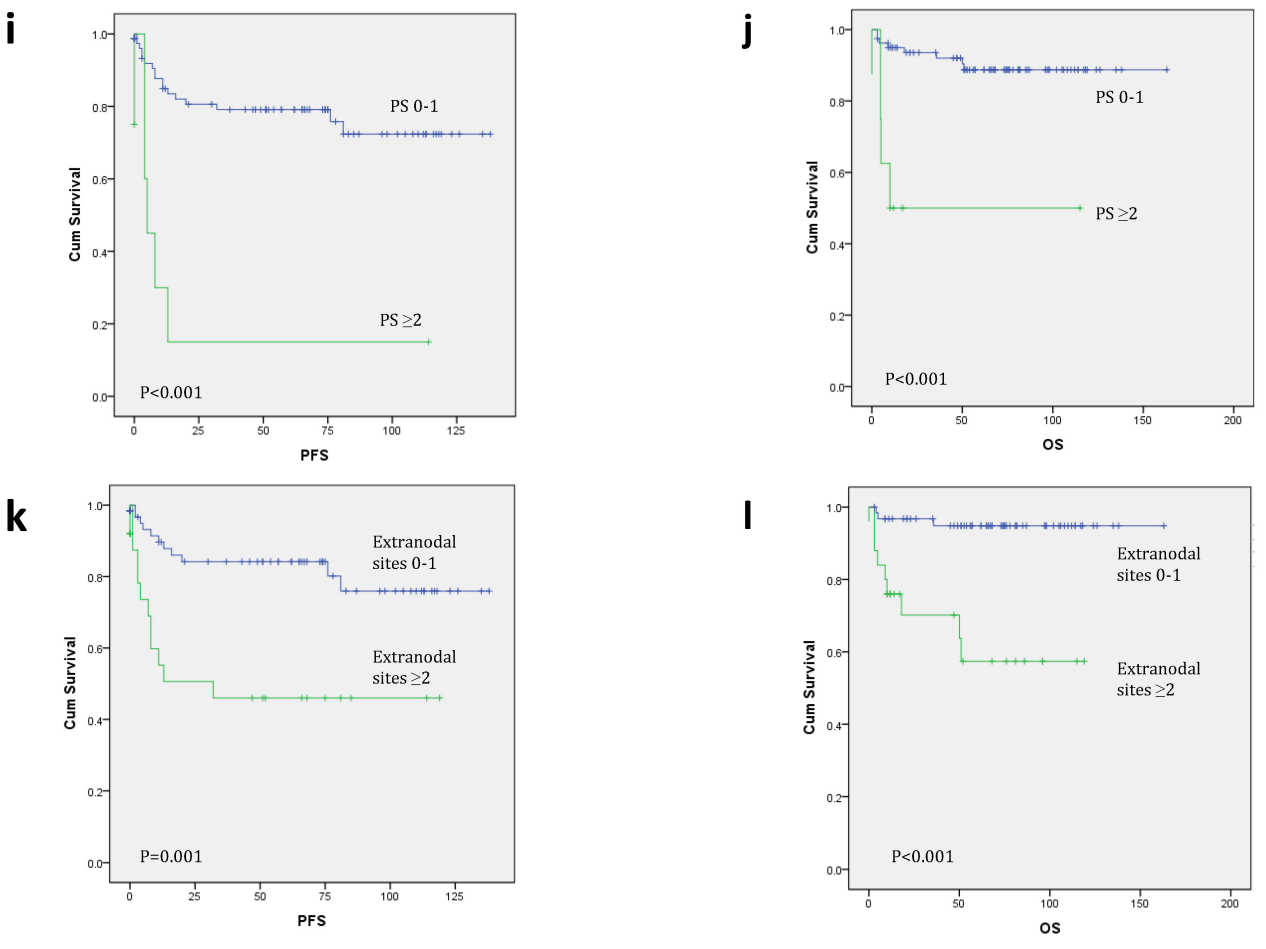
Kaplan-Meier survival curves for progression-free survival (PFS) and overall survival (OS). When analyzed by Bcl-6/Peli1 status, Bcl-6/Peli1 +/+ group showed worst PFS and OS followed by Bcl-6/Peli1 -/- group (a and b). With re-grouping as Bcl-6/Peli1 risk groups, high risk group showed worse prognosis (c and d). Other well-known prognostic factors such as high international prognostic index (e and f), high Ann Arbor stage (g and h), poor ECOG performance status (i and j) and extranodal sites (≥2) (k and l) showed worse PFS and OS.

According to nuclear expression of Peli1 and Bcl-6, there were four subgroups of Bcl-6/Peli1 status, i.e., +/+, +/-, -/+ and -/-, as shown in Figure 1. In a mechanistic viewpoint, each subgroup may indicate the following status: 1) +/+, Bcl-6 stabilized by Peli1, 2) +/-, uncoupled (independent) Bcl-6 expression despite the absence of Peli1, 3) -/+, failure of Bcl-6 expression with intact Peli1, and 4) -/-, Bcl-6/Peli1-unrelated signaling mechanism. By the pilot analysis of PFS and OS for these 4 subgroups, two favorable subgroups, i.e., +/- and -/+, and two unfavorable subgroups, i.e., +/+ and -/-, were identified (Figure 2a-b). To develop practical risk classifier, we simplified these subgroups by making two-tier Bcl-6/Peli1 risk groups composed of high risk (+/+ and -/-) and low risk (+/- and -/+). By this process, high risk group accounted for 64% (64/100) and low risk group for 36% (36/100).

### Associations between Bcl-6/Peli1 risk groups and clinicopathologic variables

Clinicopathologic associations between Bcl-6/Peli1 risk groups and clinicopathologic variables were analyzed (Table 2). Most of the conventional variables including international prognostic index (IPI) and Ann Arbor stage showed generally similar distribution. Of note, Bcl-6/Peli1 high risk group contained more frequent non-GCB subtype (83%; 53/64) by Hans classification than low risk group (64%; 23/36) (p=0.033). Among immunophenotypic markers, lack of Bcl-6 expression was also common in high risk group (69%; 44/64), compared to low risk group (28%; 10/36) (p <0.001), while other markers did not differ between the two groups.

### Survival analysis

In univariate survival analysis for PFS and OS (Table 3 and Figure 2c-l), ECOG PS (p<0.001 for both), number of extranodal sites (p=0.001 and p<0.001), Ann Arbor stage (p=0.008 and p=0.006) and IPI (p<0.001 for both) were significant prognostic factors, as well as B symptoms for OS (p=0.030). Bcl-6/Peli1 risk group also predicted prognosis for PFS and OS (p=0.026 and p=0.021).

In multivariate analysis for PFS with all significant variables observed in univariate analysis (Table 3), Bcl-6/Peli1 risk group (p=0.032, HR=3.29) was observed as an independent prognostic factor, along with IPI (p=0.013, HR=3.39) and ECOG PS (p=0.035, HR=3.08). In multivariate analysis for OS, Bcl-6/Peli1 risk group (p=0.048, HR=7.87) and IPI (p=0.001, HR=12.15) were significantly associated with prognosis.

## Discussion

In the present study, we investigated the prognostic utility of Peli1 expression in association with Bcl-6 status, and observed Bcl-6/Peli1 risk group as an independent prognostic indicator for PFS and OS.

Since standard immunochemotherapy with R-CHOP regimen has been established in DLBCL, various methods for risk stratification have been developed due to its heterogeneous outcome 13, as shown in 'cell of origin' classification by using gene expression profiling or immunohistochemical algorithm 11. In many studies, non-GCB or activated B cell-like (ABC) subtype predicts poor prognosis compared to GCB subtype 11, while proportion of non-GCB subtype and its prognostic value variably depends on geographic location, detection methods and various heterogeneous subset within each subtype 14-16.

*BCL6* gene was known to function in germinal center (GC) formation during immune reaction. It exerts its role as a powerful transcriptional repressor targeting DNA damage sensing by *TP53* and* ARF* and escape process from the GC reaction toward plasma cell differentiation by *IRF4* and *PRDM1*, allowing sufficient time to get somatic hypermutation in immunoglobulin genes 17. Deregulated Bcl-6 expression with disruption of many signals leads to lymphomagenesis in mice 18. Among various deregulation mechanism leading to maintained expression of Bcl-6 17, 19-21, ubiquitin-mediated stabilization is a feasible mechanism for sustained expression of nuclear factors 22, 23.

Prognostic effects of Bcl-6 protein are relatively complex in DLBCL. *BCL6* itself is an oncogene of DLBCL. At the same time, Bcl-6 protein is a component of GCB marker, while it can also be variably expressed in non-GCB subtype and influences many other critical signaling pathways 24. In many studies with clinical samples, Bcl-6 was observed as a good prognostic factor in DLBCL 25, 26. From the previous mechanistic report 6, we focused on the functional relevance of Peli1 and Bcl-6, a known target of Peli1-induced lymphomagenesis. With nuclear co-localization status of both molecules, we divided two major prognostic groups, i.e., high risk and low risk groups.

In our clinicopathologic analysis, this risk modeling worked well as a significant prognostic indicator independently from IPI and other conventional variables. Considering that GCB and non-GCB subtypes contain heterogeneous prognostic subsets, our mechanism-based approach to dissect Bcl-6 expression according to E3 ligase Peli1 led to redefined clinical utility for risk stratification in addition to Bcl-6 or 'cell of origin' classification. It is of note that Bcl-6-/Peli1- subgroup was very aggressive nearly similar to Bcl-6+/Peli1+ subgroup, constituting high risk group in our risk model. Bcl-6-/Peli1- subset may largely belong to non-GCB subtype (41/44, 93%) and therefore, may be driven by Bcl-6-independent genetic alteration for non-GCB subtype. It remains to be clarified further to determine the detailed molecular mechanism of each subgroup, as well as clinical applicability via validation cohort.

A few previous studies addressed consistent results similar to our study 6, 8, 27, but with significant differences. Two previous studies focused on mechanism of Peli1-mediated cell survival or lymphomagenesis 6, 27. In the studies for prognostic effects of Peli1 in DLBCL 6, 8, the interpretation criteria and prognostic significance were slightly different from our study. In the study by Park et al, DLBCL with ≥10% of cells showing nuclear expression of moderate to strong intensity were defined as Peli1^hi^, and otherwise Peli1^lo^. Peli1^hi^ group had a worse prognosis than Peli1^lo^ group for OS 6. In their study, the prognostic significance of Peli1 for OS was independent of international index (IPI) in all DLBCL patients (p=0.010 for Peli1; p<0.001 for IPI), but not in R-CHOP-treated patients in multivariate analysis for OS (p=0.073 for Peli1; p=0.015 for IPI). In the setting of prognostic analysis, the meaning of Peli1 expression was not interpreted according to Bcl-6 expression status, while their expression levels showed a positive correlation in the Bcl-6-translocation-negative subset of DLBCL. In the study by Choe et al, they showed that Peli1>2 group was a significant prognostic indicator only for relapse-free survival (RFS) in univariate analysis (p=0.002), but not for OS or in multivariate analysis (p=0.591) 8. In their study, the prognostic significance of Peli1 was not analyzed according to Bcl-6 expression status. In the present study, we focused on the close mechanistic relationship between Peli1 and Bcl-6, and the clinical effects of Peli1 expression according to expression status of Bcl-6 to make a clinically relevant indicator. Our survival analysis showed that Bcl-6/Peli1 risk group was significantly associated with prognosis for both PFS and OS in multivariate analysis, independently from IPI and conventional variables.

With the limitation of retrospective design of this analysis, our data suggest that combined interpretation of the two related molecular expression, Bcl-6 and Peli1, may harbor significant clinical implication. Validation study with larger independent cohort is needed to apply this model to clinical risk stratification.

In summary, we composed a new risk model of Bcl-6/Peli1 by using E3 ligase Peli1 and its known target, Bcl-6 in the basis of lymphomagenesis, and this model independently predicted prognosis in DLBCL patents for PFS and OS. With proper validation, it may contribute to risk assessment in patients with DLBCL.

## Supplementary Material

Supplementary figure 1: enrollment criteria.

## Figures and Tables

**Table 1:** Clinicopathologic characteristics of diffuse large B cell lymphoma

Clinicopathologic characteristics		No. of cases (%)
Age	≤60	48/100 (48%)
	>60	52/100 (52%)
Sex	Female	43/100 (43%)
	Male	57/100 (57%)
B symptoms	Absent	79/100 (79%)
	Present	21/100 (21%)
ECOG PS	<2	91/100 (91%)
	≥2	9/100 (9%)
Serum LDH^†^	Normal	49/97 (51%)
	Elevated	48/97 (49%)
Ann Arbor stage	Ⅰ-Ⅱ	52/100 (52%)
	Ⅲ-Ⅳ	48/100 (48%)
Number of extranodal sites	<2	73/100 (73%)
	≥2	27/100 (27%)
International prognostic index	0-2	65/100 (65%)
	3-5	35/100 (35%)
Bone marrow involvement^†^	Absent	79/92 (86%)
	Present	13/92 (14%)
Bulky disease (>10cm)	<10	91/100 (91%)
	≥10	9/100 (9%)
Hans classification	GCB	24/100 (24%)
	Non-GCB	76/100 (76%)
BCL2 expression	Negative	36/100 (36%)
	Positive	64/100 (64%)
BCL6 expression	Negative	54/100 (54%)
	Positive	46/100 (46%)
CD10 expression	Negative	85/100 (85%)
	Positive	15/100 (15%)
MUM1 expression	Negative	35/100 (35%)
	Positive	65/100 (65%)
Peli1 nuclear expression	Negative	70/100 (70%)
	Positive	30/100 (30%)
Bcl-6/Peli1 combination	Negative/Negative	44/100 (44%)
	Positive /Negative	26/100 (26%)
	Negative/Positive	10/100 (10%)
	Positive/Positive	20/100 (20%)
Treatment	R-CHOP	88/100 (88%)
	R-others	12/100 (12%)
Total N (%)		100 (100%)

ECOG PS, Eastern Cooperative Group Performance Status; LDH, lactate dehydrogenase; GCB, germinal center B cell-like; R-CHOP, rituximab, cyclophosphamide, doxorubicin, vincristine and prednisone. ^†^These variables excluded missing values.

**Table 2:** Associations between Bcl-6/Peli1 risk groups and clinicopathologic variables in diffuse large B cell lymphoma.

Clinicopathologic characteristics		Bcl-6/Peli1 risk group		
		High risk	Low risk	P value
Age	≤60	32/64 (50%)	16/36 (44%)	0.594
	>60	32/64 (50%)	20/36 (56%)	
Sex	Female	27/64 (42%)	16/36 (44%)	0.827
	Male	37/64 (58%)	20/36 (56%)	
B symptoms	Absent	51/64 (80%)	28/36 (78%)	0.822
	Present	13/64 (20%)	8/36 (22%)	
ECOG PS	<2	57/64 (89%)	34/36 (94%)	0.482*
	≥2	7/64 (11%)	2/36 (6%)	
Serum LDH^†^	Normal	30/62 (48%)	19/35 (54%)	0.577
	Elevated	32/62 (52%)	16/35 (46%)	
Ann Arbor stage	Ⅰ-Ⅱ	34/64 (53%)	18/36 (50%)	0.764
	Ⅲ-Ⅳ	30/64 (47%)	18/36 (50%)	
Number of extranodal sites	<2	45/64 (70%)	28/36 (78%)	0.420
	≥2	19/64 (30%)	8/36 (22%)	
International prognostic index	0-2	40/64 (63%)	25/36 (69%)	0.485
	3-5	24/64 (27%)	11/36 (31%)	
Bone marrow^†^ involvement	Absent	50/58 (86%)	29/34 (85%)	1.000*
	Present	8/58 (14%)	5/34 (15%)	
Bulky disease (>10cm)	<10	57/64 (89%)	34/36 (94%)	0.482*
	≥10	7/64 (11%)	2/36 (6%)	
Hans classification	GCB	11/64 (17%)	13/36 (36%)	0.033
	Non-GCB	53/64 (83%)	23/36 (64%)	
BCL2 expression	Negative	21/64 (33%)	15/36 (42%)	0.376
	Positive	43/64 (67%)	21/36 (58%)	
BCL6 expression	Negative	44/64 (69%)	10/36 (28%)	<0.001
	Positive	20/64 (31%)	26/36 (72%)	
CD10 expression	Negative	56/64 (88%)	29/36 (81%)	0.351
	Positive	8/64 (12%)	7/36 (19%)	
MUM1 expression	Negative	21/64 (33%)	14/36 (39%)	0.541
	Positive	43/64 (67%)	22/36 (61%)	
Treatment	R-CHOP	55/64 (86%)	33/36 (92%)	0.529*
	R-others	9/64 (14%)	3/36 (8%)	
Total N (%)		64 (100%)	36 (100%)	

ECOG PS, Eastern Cooperative Group Performance Status; LDH, lactate dehydrogenase; GCB, germinal center B cell-like; R-CHOP, rituximab, cyclophosphamide, doxorubicin, vincristine and prednisone.^ †^These variables excluded missing values. P values were calculated by Pearson's chi-square test (2-sided) or Fisher's exact test (2-sided).^*^

**Table 3:** Survival analysis with Bcl-6/Peli1 risk groups and clinicopathologic variables in diffuse large B cell lymphoma with R-CHOP treatment.

Clinicopathologic variables		Progression-free survival			Overall survival		
		Univariate analysis	Multivariate analysis		Univariate analysis	Multivariate analysis	
		P value	P value	HR [95% CI]	P value	P value	HR [95% CI]
Age	>60	0.068			0.064		
Sex	Male	0.940			0.870		
ECOG PS	≥ 2	<0.001	0.035	3.08 [1.08-8.78]	<0.001	0.169	
B symptoms	Present	0.226			0.030	0.349	
Serum LDH	Elevated	0.101			0.839		
Number of extranodal sites	≥ 2	0.001	0.595		<0.001	0.127	
International prognostic index	3-5	<0.001	0.013	3.39 [1.30-8.86]	<0.001	0.001	12.15 [2.64-55.89]
Bone marrow involvement	Present	0.529			0.551		
Bulky disease	≥ 10cm	0.172			0.909		
Ann Arbor stage	III-IV	0.008	0.648		0.006	0.532	
Hans classification	Non-GCB	0.642			0.912		
Bcl-2 expression	Positive	0.340			0.783		
Bcl-6 expression	Positive	0.605			0.708		
CD10 expression	Positive	0.931			0.926		
MUM1 expression	Positive	0.252			0.355		
Bcl-6/Peli1 risk group	High risk	0.026	0.032	3.29 [1.11-9.71]	0.021	0.048	7.87 [1.01-62.50]

R-CHOP, rituximab, cyclophosphamide, doxorubicin, vincristine and prednisone; ECOG PS: the Eastern Cooperative Group Performance Status; LDH: lactate dehydrogenase; GCB, germinal center B cell-like; HR, hazard ratio; 95% CI, 95% confidence interval.
