# Supplementary figures and images for: Bcl-6-dependent risk stratification by nuclear expression of Peli1 in diffuse large B-cell lymphoma
Source: J Cancer. 2022 Nov 14;13(15):3598–605. doi: 10.7150/jca.67569 (PMC9809313; doi:10.7150/jca.67569)

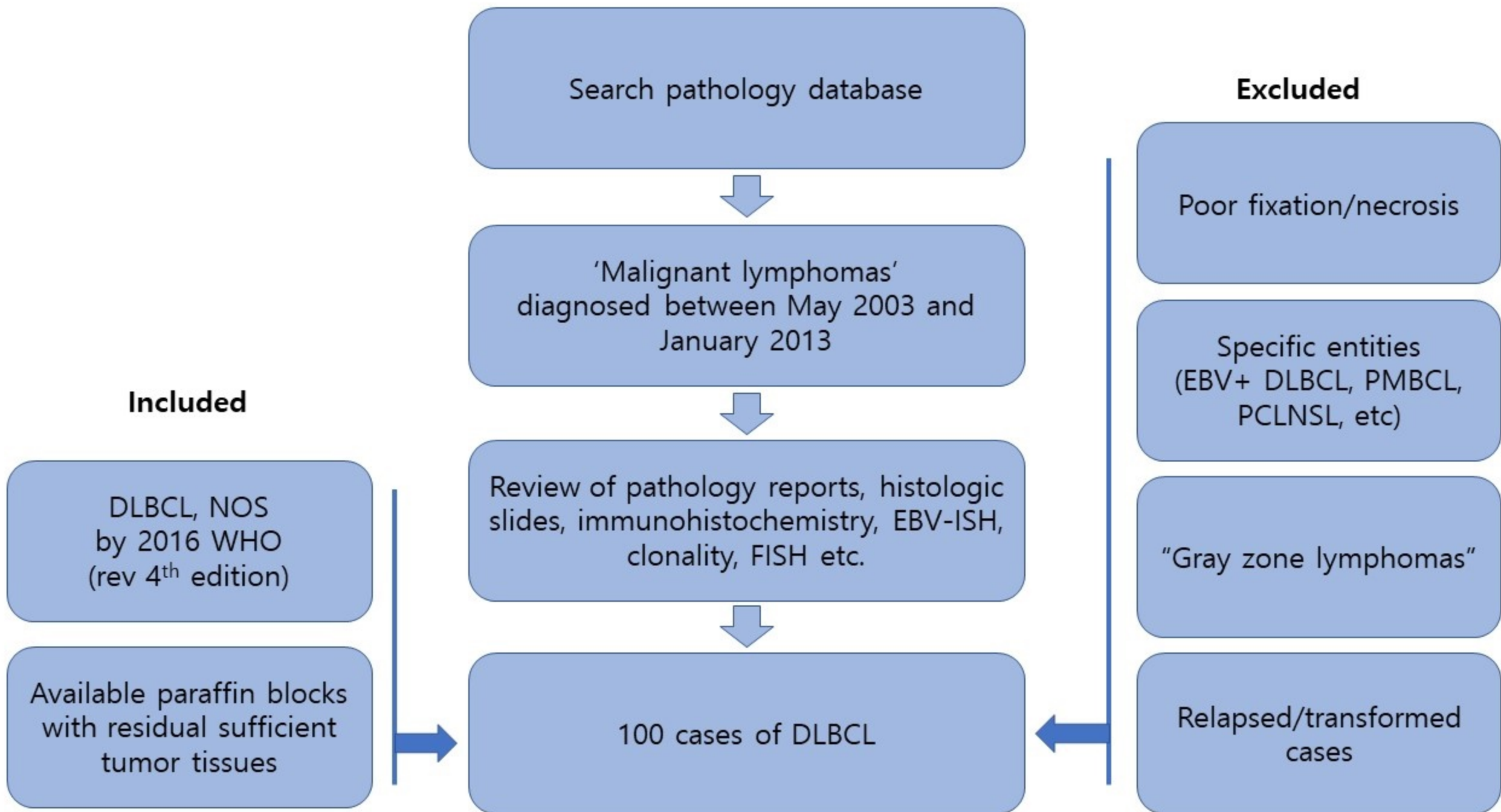

Supplement: Supplementary file 1 — Supplementary figure 1: enrollment criteria. [file jcav13p3598s1.pdf]
